# The intersection of undernutrition, microbiome, and child development in the first years of life

**DOI:** 10.1038/s41467-023-39285-9

**Published:** 2023-06-15

**Authors:** Fanette Fontaine, Sondra Turjeman, Karel Callens, Omry Koren

**Affiliations:** 1 Food and Agriculture Organization of the United Nations, Rome, Italy; 2 Université Paris- Cité, 75006 Paris, France; 3 Azrieli Faculty of Medicine, Bar-Ilan University, Safed, Israel

**Keywords:** Microbiology, Nutrition

## Abstract

Undernutrition affects about one out of five children worldwide. It is associated with impaired growth, neurodevelopment deficits, and increased infectious morbidity and mortality. Undernutrition, however, cannot be solely attributed to a lack of food or nutrient deficiency but rather results from a complex mix of biological and environmental factors. Recent research has shown that the gut microbiome is intimately involved in the metabolism of dietary components, in growth, in the training of the immune system, and in healthy development. In this review, we look at these features in the first three years of life, which is a critical window for both microbiome establishment and maturation and child development. We also discuss the potential of the microbiome in undernutrition interventions, which could increase efficacy and improve child health outcomes.

## Abstract (web summary)

In this review article, the authors provide an overview of the interrelationships between the microbiome and nutrition in child growth and healthy development and discuss the potential of the microbiome in undernutrition interventions.

## Introduction

Undernutrition remains one of the most pressing global health challenges today, affecting mainly infants and children. It comes in different forms: wasting (low weight-for-height, usually a result of severe and acute weight loss), stunting (low height-for-age, reflecting chronic or recurrent undernutrition), and being underweight as well as deficiencies in vitamins and minerals. In 2020, there were still an estimated 149 million children under the age of 5 years exhibiting stunted growth and an estimated 45 million who were wasted^1^. Cases mostly occur in low- and middle-income countries (LMIC) from sub-Saharan Africa and Southi. Low birth weight also remains a problem even though the situation seems to be improving with the global prevalence decreasing from 17.5% in 2000 to 14.6% in 2015.

Undernutrition contributes to nearly half of deaths in children under 5 years of age^2^; it weakens the immune system, increasing the risk of death from infectious diseases. Consequences of undernutrition impact all areas of development (growth, neurodevelopment, metabolic). Importantly, children are not immune to the “dual burden” of malnutrition (i.e., undernutrition and obesity). The double burden can manifest throughout one’s lifespan. Childhood stunting has long-lasting physiologic effects and is associated with increased risk for adult diseases such as obesity, cardiovascular diseases, and diabetes^1^.

Steady progress has been made to reduce stunting since 2000 in most regions of the world, but it remains insufficient. In addition, recent and ongoing global crises are expected to exacerbate food insecurity and poverty, compromising the efforts to achieve the United Nations Sustainable Development Goals 2 and 3 by 2030: Goal 2: “End hunger, achieve food security and improve nutrition” and Goal 3: “Ensure healthy lives and promote well-being for all at all ages”.

Studies have emphasized that undernutrition cannot be ascribed solely to food security, and rather reflects the intersection of multiple factors, among which carriage of pathogens and impairment of intestinal functions observed in environmental enteropathies are key. Extra energy and nutrients are necessary, but not always sufficient, to reverse undernutrition. In children with stunting, only about 10% of the linear growth deficit is corrected by nutritional supplementation. Interventions on water quality, sanitation, handwashing, and nutrition have also shown limited impact on diarrhea and child growth^3–5^, suggesting that the roles of other biological pathways may have been underestimated.

A growing body of evidence shows that the microbiome, the billions of microorganisms present in our body, and particularly, in our gut, is intimately involved in weight gain and loss and immune system training and modulation as well as in overall host homeostasis^6–9^. The human microbiome is shaped by various factors such as genetics, age, sex, and mostly, the environment, including geography, mode of birth, medication, and lifestyle^10–12^. Thus, the microbiome appears to be an important biological player mediating the impact of environmental factors on child development and health^13^. Numerous studies demonstrated that early life events, and in particular nutrition, are critical for the acquisition (at birth) and development (until the age of three years) of the gut microbiome, which contributes to healthy development.

The purpose of this review is to explore the relationship between the microbiome and undernutrition and micronutrient deficiencies in the first years of life and discuss how knowledge about the gut microbiome could help to improve child undernutrition prevention and treatments.

## Development of the child microbiome

Development of the infant gut microbiome is via de novo assembly of a complex microbial community that is influenced by maternal and infant factors as well as environmental factors, but that follows some predictable patterns. The colonization process of the infant gut microbiome begins mostly at birth, by vertical transmission from multiple maternal body sites (skin, vagina, oral cavity, and mostly gut)^14,15^. The first gut colonizers, facultative anaerobic bacteria belonging to the phyla Proteobacteria (e.g., *Escherichia*, *Enterobacter*, *Enterococcus*) and Firmicutes (*Staphylococcus*, and *Streptococcus*), shape the early environment, which influences the dynamic succession of subsequent microbes, mostly obligate anaerobes, like *Bifidobacterium*, *Bacteroides*, and *Clostridium*^16^. *Bifidobacterium* abundance starts to increase around 3–4 days after birth and becomes a predominant genus at around one month^17,18^. The set of bacterial species that dominate the gut from the first hours after birth usually prevail during the first month of life^17^, even if colonization stability is greatly influenced by the mode of birth^19^. Infants also continue to acquire a vast array of microbes from their mother and their environment.

Microbiome maturation is strongly modulated by age and development stage, including changes to infant diet with age. In the first weeks and months of life, the microbiome evolves slowly as infant diet is homogeneous. Breastfeeding is considered one of the most significant factors associated with neonate microbiome structure^20,21^. The most remarkable feature is the increased level of Bifidobacteria in breastfed babies. The introduction of complementary food and/or interruption of breastfeeding induces major changes in the bacterial population from a breast milk-adapted gut microbiota toward increased diversity and major metabolic changes. As an infant ages, the proportion of complementary food in the diet increases, further modifying the gut microbiome. A longitudinal study of the gut microbiome of 903 infants from four high-income countries, Germany, Finland, Sweden, and the US, during the first three years of life, revealed three distinct phases in microbiome development with respect to both composition and function^22^. The first phase of development, from months 3–14, the developmental phase, corresponds to the introduction of the first solid foods. It is first dominated by *Bifidobacterium* *spp.*, but the relative abundance of the five major phyla, Actinobacteria, Bacteroidetes, Firmicutes, Proteobacteria, and Verrucomicrobia, shift and alpha diversity increases. From months 15–30, a transitional phase is characterized by a major increase in Bacteroidetes and a decrease in Proteobacteria. In the same period, alpha diversity continues to increase. In the stable phase from 31 to 46 months, both composition and alpha diversity remain largely unchanged. This is also in line with other studies^16,23,24^.

These trends reflect the main dynamics of the gut microbiota of healthy infants in industrialized countries but are not universal^25^. During the first six months of life, all infant populations across lifestyles are dominated by *Bifidobacterium* and *Streptococcus*, but with divergence in species and gene contents (particularly associated with human milk oligosaccharide (HMO) degradation). *Bifidobacterium breve,* with limited HMO degradation abilities, is more abundant in industrialized populations while *Bifidobacterium longum* subsp*. infantis (B. infantis)*, dominates in traditional lifestyles/LMICs. After six months, the guts of infants living non-industrialized or transitional lifestyles become enriched with *Prevotella* and *Faecalibacterium* as well as an important number of uncharacterized species (up to 20% in hunter-gatherer populations) that are absent in infants from industrialized environments (dominated by *Bacteroides*)^26^. Interestingly, the gut microbiome of infants living transitional lifestyles (e.g., Malawi, South Africa, India, Peru) showed intermediate phenotypes between those of non-industrialized (hunter-gatherer in Tanzania and Bassa in Nigeria) and industrialized infants^26^. However, there are little data on healthy microbiome development in LMICs, including in areas where undernutrition is prevalent, compared to the HIC context.

Gut microbiome development is intimately linked with the development of the child. Disturbances in host-microbe coevolution and optimal microbial succession could impact immune functions, intestinal development, nutritional energy harvest, neurodevelopment, growth, and weight gain. As an example, in a Swedish cohort, children with slower than expected weight gain (reference curve between 12 months and 5 years of age) were characterized by lower microbial diversity and reduced abundance of *Faecalibacterium* and *Ruminococcus* at 12 months compared with children with normal or faster weight development^27^.

Interestingly, critical modifications of the gut microbiome arise when the diet evolves from an exclusive milk diet to one that includes complementary foods in the first months or years of life. The period is also critical for the nutritional status of the child. Lack of food or inappropriate feeding practices, in addition to higher exposure to pathogens, affect the microbiome with potential impacts on child development. Changes in the composition and maturity of the gut microbiome could correlate with or even contribute to undernutrition.

## Undernourished child microbiome

Several studies have identified differences between healthy and undernourished child microbiomes. In Bangladesh, the gut microbiota composition of acutely undernourished infants was found to differ significantly from that of healthy babies, with a dramatic increase in the abundance of Proteobacteria, including pathogenic genera (such as *Klebsiella*, *Escherichia*, *Shigella*, and *Streptococcus*). There were also minimal levels of Bacteroidetes and reduced alpha diversity^28^. In another study, in 3- to 24-month-old Bangladeshi infants with severe acute malnutrition (SAM), the absolute abundance of the early colonizer *B. infantis*, with a metabolic capacity to use a wide range of HMOs, was found to be lower compared to healthy age-matched counterparts^29^. Million et al.^30^, who studied children suffering from SAM in five different African and Asian countries, reported depletion of several species of the Bacteroidaceae, Eubacteriaceae, Lachnospiraceae, and Ruminococcaceae families and the methanogenic archaeal species *Methanobrevibacter smithii*, while potential pathogens, e.g., *Enterococcus faecalis*, *E. coli*, and *Staphylococcus aureus* were consistently enriched^30^. The importance of *M. smithii*, a key player in energy harvest, was also confirmed in another study in Mali in which SAM was also highly associated with the loss of this taxa ^31^. In a study of Malawian twin pairs discordant for Kwashiorkor, a form of SAM, Kwashiorkor was also associated with Proteobacteria and Fusobacteria bloom^32^. In a study on a rural Gambia cohort, children with Marasmus (another form of SAM) had distinct microbiome characteristics (lower microbial richness and biomass, enrichment in Enterobacteriaceae, altered interactions between specific Enterobacteriaceae) not observed among children with moderate acute malnutrition (MAM)^33^. Stunted children also harbored distinct phage populations compared to healthy counterparts, with those isolated from stunted children favoring Proteobacteria growth^34^.

Beyond the microbiota composition measured at important developmental windows, overall maturation of the microbiome is critical for healthy child development and growth. Using longitudinal studies on cohorts of children from Bangladesh and Malawi with normal growth or suffering from undernutrition, machine-learning models were applied to identify bacterial taxa that were the most discriminant for healthy growth, by age bracket. Indicators created for infant gut microbiota development were the “relative microbiota maturity index” and “microbiota-for-age *z*-score” (MAZ), similar to the anthropometric *z*-scores used to assess nutritional status^32^. In undernourished children, the MAZ score was low as maturation was severely impaired. The most discriminatory species between healthy and undernourished children were *B. longum* in the first six months of life, and then *Faecalibacterium prausnitzii*, *Ruminococcus* species, and *Dorea* species from 6–24 months of age^35–37^. Other models have integrated functional pathways to assess microbiome age and host state^36,38^. In Malawi, infants with Kwashiorkor had a functionally immature microbiome along with disruptions in carbohydrate and amino acid metabolism^38^. In a Zimbabwe cohort, functional metagenomic features, particularly vitamin B and nucleotide biosynthesis pathways, better predicted child growth than taxonomic microbiome features^39^. Notably, microbiome development is complex and is not restricted to a succession of discrete taxa but also their community dynamics. A recent longitudinal study on healthy Bangladeshi children from 1–60 months old assessed the interactions between gut microbiome members over time^40^. A group of 15 covarying bacterial taxa was identified, called an *ecogroup*. It provided a concise description of microbiota colonization and could also be applied to describe gut microbiome development in two other cohorts of healthy children in Peru and India^40^.

Additionally, in a Malawian cohort of children from 6–30 months old, gut microbiome maturity was inversely correlated with inflammation, intestinal permeability, damage, and biomarkers for environmental enteric dysfunction (EED). The presence of these biomarkers was predicted by several *Bifidobacterium* and Enterobacteriaceae taxa as well as displaced oral taxa^41^. The small intestinal microbiome has been less studied due to the difficulty of accessing duodenal fluids without invasive esophagogastroduodenoscopy, but available data suggest that it could also contribute to malnutrition, in particular undernutrition, in the context of EED. In a cohort of Bangladeshi undernourished children with EED, 14 taxa (which are not typically classified as enteropathogens) were negatively correlated with linear growth and positively correlated with duodenal proteins involved in immune inflammatory responses^42^. Some of these taxa, including species from the genera *Veillonella*, *Streptococcus*, *Haemophilus*, and *Neisseria*, were also overrepresented in small intestine samples of stunted children in sub-Saharan Africa (Madagascar and Central African Republic)^43^. Small intestinal bacterial overgrowth is commonly found in stunted children and is often characterized by an overgrowth of oral bacteria. This ectopic colonization of the oral microbiome in the small intestine might be involved in undernutrition as it has been shown to disturb the absorption of lipids in experimental models^44^.

Notably, studies showed that changes associated with undernutrition are transferable via fecal microbial transplantation (FMT) into animal models, suggesting a causal role of the microbiome in stunting and enteropathy outcomes. Germ-free (GF) mice that received FMT from malnourished Malawian children gained substantially less weight and showed impaired growth^37^ as well as diet-induced enteropathy^45^ when compared with those that received transplants from healthy children. Complex dynamics in the microbial population seem to be involved, with both loss of growth-supporting species and an increase in pathobionts. Indeed, *Ruminococcus gnavus* and *Clostridium symbiosum* were identified as the main bacteria responsible for the weight gain seen in mice receiving FMT from well-nourished Malawian children, with both able to ameliorate the impaired growth phenotype transmitted to the mice via undernourished donor microbiota^37^. In another study, data suggested that during undernutrition, cross-feeding between intestinal pathobionts (Enterobacteriaceae and Bacteroidales species) promoted their overgrowth^46^. In addition, duodenal strains obtained from children with enteropathy transferred to GF mice induced enteropathy in the animals’ small intestines^42^.

Infections also seem to play an important role in the undernutrition phenotype. In the first two years of life, high carriage of enteropathogens is inversely associated with both ponderal and linear growth. Pathogens could disturb the maturation of the gut microbiome, or conversely, the implantation as well as the expression of virulence genes of pathogenic bacteria could be modulated by the larger intestinal community. Current data support the second hypothesis. An enterotoxigenic strain of *Bacteroides fragilis* (ETBF) from a stunted child was transferred to mice together with microbiomes from healthy or undernourished children. ETBF induced weight loss only when introduced on the background of an unhealthy bacterial community^47^.

**Fig. 1:**
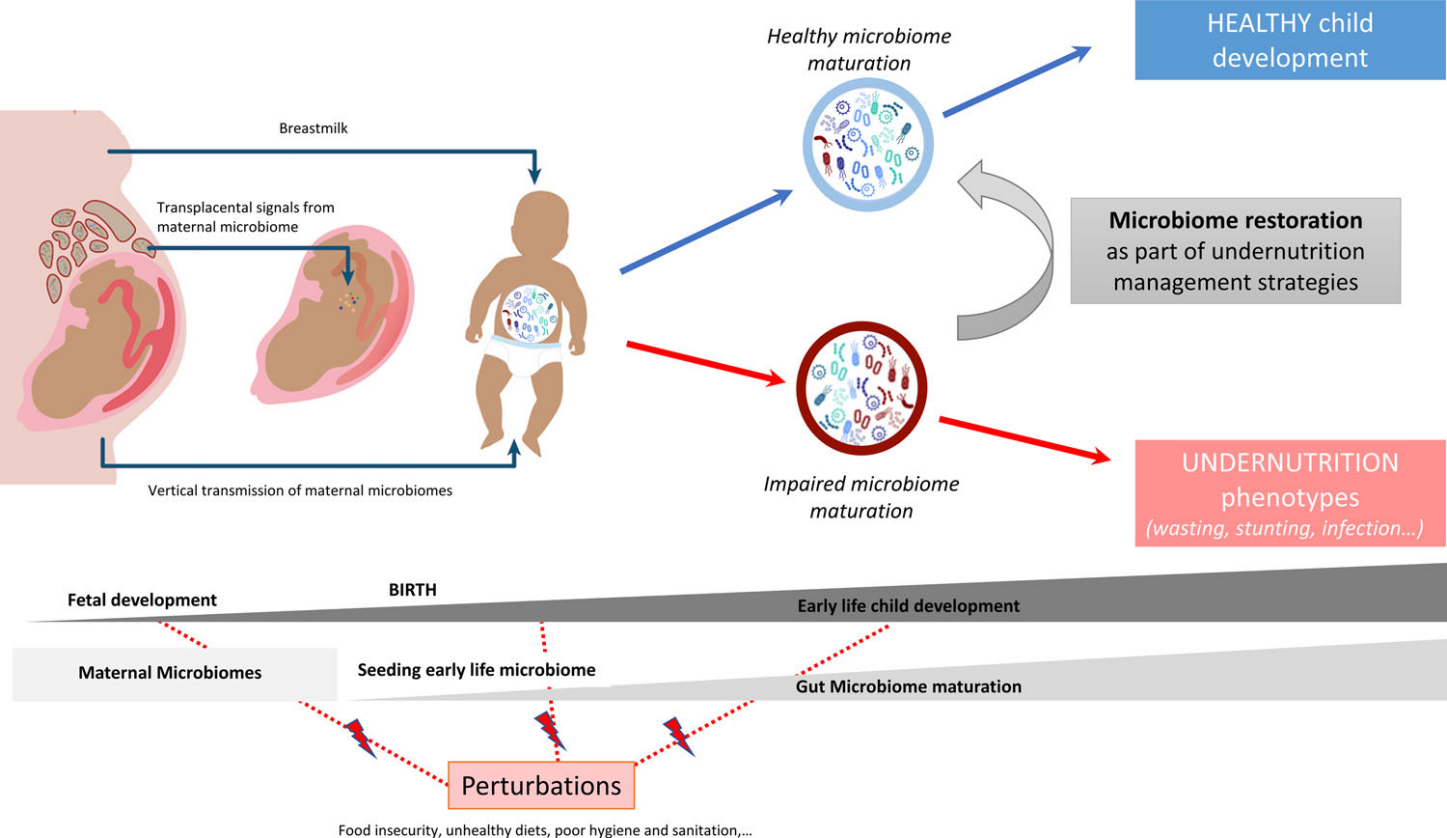
The gut microbiome and child undernutrition phenotypes. Undernutrition seems to have intergenerational origins. Part of the intergenerational effects of undernutrition could be mediated by the microbiome through transplacental signals during pregnancy that could influence fetal development, vertical transmission of the microbiome at birth, and then through breastfeeding and regular skin contact in early life. A healthy developmental trajectory of the child gut microbiome supports healthy child development. Perturbations, such as food insecurity, unhealthy diets, and poor hygiene and sanitation impact maternal and child health and can lead to undernutrition phenotypes (e.g., stunting, wasting, and micronutrient deficiencies). Some of these effects seem to be mediated by an impaired gut microbiome. Microbiome restoration through food and microbial interventions could help to improve undernutrition management strategies and improve child development and health. Figure inspired by refs. ^13, 93^.

Altogether, these results suggest that disturbed and immature gut and duodenal microbiomes are associated with undernutrition and may also play a causal role in the diverse manifestations of undernutrition: mostly on low weight gain but also on infection and potentially reduced growth (Fig. 1). The use of biomarkers/indices to evaluate child microbiome maturity could help to characterize undernourished states and to evaluate intervention effectiveness in treating undernutrition. Accordingly, restoration of the microbiome might need to be considered for better prevention and treatment of undernutrition.

## Undernutrition management in the context of the microbiome

There is no consistent guidance on how best to manage children with MAM, as there is a paucity of intervention studies. Supplementary foods and nutrition counseling are usually proposed^48^. The management of SAM usually involves both broad-spectrum antibiotics and therapeutic foods (locally produced or standardized as ready-to-use therapeutic food (RUTF))^49^.

### Antibiotics

Antibiotics exhibit growth-promoting effects, which may be mediated, in part, by treatment of clinical or subclinical infections, more common among malnourished children^50^. Nevertheless, in light of microbiome data and, despite being life-saving medications, antibiotic use might also have counterproductive effects. In Burkina Faso, in children with uncomplicated SAM, there was no reduction in microbiome alpha diversity 8 weeks after treatment with amoxicillin or azithromycin, but azithromycin significantly increased the abundance of potentially pathogenic bacteria that can cause invasive diarrhea (*Salmonella* and *Shigella* spp.)^51^. In addition, a recent study found that antibiotic use in the neonatal period actually stunted male growth in childhood, and a fecal transplant from the neonates to mice demonstrated the same phenotype in the mice^52^. In mice, treatment with cotrimoxazole, recommended as prophylactic treatment by the World Health Organization (WHO) for undernourishment, does not restore the gut microbiome toward that of healthy controls but instead induces a distinct dysbiosis (reduction of butyrate producers *Faecalibacterium* and *Anaerotruncus*)^53^. More longitudinal studies in humans are needed to unravel the impact of antibiotic treatment on the microbiome and in various contexts (or forms) of undernutrition.

### Therapeutic foods

In children with SAM, therapeutic food interventions have been successful in reducing mortality in children with SAM but insufficient, in some cases, for restoring healthy growth^35^. This may be partly related to microbiome maturation. A short study in Uganda on children with SAM showed that the introduction of a safe and cost-effective legume-enriched product containing fermentable carbohydrates (cowpea) as a complementary food had a positive impact on the gut microbiome (higher Bifidobacteria), compared to other prebiotic supplements, like inulin^54^. However, in Bangladesh, traditional therapeutic foods, such as Khichuri-halwa administered together with milk-suji, were used to treat SAM but did not support microbiome restoration or maturity^35^. In contrast, a commercially available RUTF, a nutrient-packed peanut paste fortified with vitamins and minerals, has changed the treatment of undernourished children (with SAM or Kwashiorkor). Studies showed that this RUTF improved the functional maturity of the gut microbiome for the first six months following its initiation; however, this improvement was not sustained over time^35,36,38^.

Knowledge of gut microbiome maturation offers an opportunity to design and test optimal therapeutic foods that steer the microbiota toward an age-appropriate, healthy state. Gehrig et al.^36^ characterized gut microbial communities and host responses over the course of 12 months in Bangladeshi children who were treated for SAM with one of three conventional therapeutic foods (imported RUTF PlumpyNut from Nutriset, or two locally produced therapeutic foods, a rice-lentil formulation and a chickpea-containing formulation). They then tested the effect of microbiota-directed complementary food (MDCF) prototypes targeting bacterial taxa underrepresented in SAM and MAM microbiota in animal models. They identified 3 MDCFs, and one of these (MDCF-2) gave encouraging preliminary results in clinical trials in children with MAM^36^. A second clinical trial also evaluated the effects of MDCF-2 (a combination of four ingredients: chickpea, peanut, banana, and soy flour) versus a rice-lentil Ready-to-Use Supplementary Food (RUSF) to treat MAM in Bangladesh. MDCF-2 was similar in energy density and micronutrient content to the RUSF and met all other safety and WHO requirements for complementary/supplementary food for 12- to 18-month-old children with MAM. After the 3-month intervention, children in the MDCF-2 group had better outcomes than those in the RUSF group with respect to the mean change in key anthropometric measurements (weight-for-length and weight-for-age z-scores) as well as larger changes in plasma protein mediators of bone growth, neurodevelopment, and inflammation. In addition, there was a complete repair of the gut microbiota in children who received MDCF-2 than in those who received the RUSF. Following MDCF-2 consumption, the greatest bacterial increases occurred for *P. copri* and *F. prausnitzii*, and the greatest decreases were in *Bifidobacterium* spp. (likely *B. longum*), which is a hallmark of successful gut-community development^55,56^. Longer follow-up is needed to assess the durability of the effects and their relationship with child physiological parameters, but early data suggest that MDCFs represent a major advance in the treatment of MAM and could be more widely implemented (Fig. 1).

### Probiotics

Some probiotics may help to improve child growth through the prevention of infections and micronutrient and vitamin deficiencies. In addition, specific probiotic strains might be used to “restore” depleted bacteria that are critical for the gut microbiome development of children suffering from undernutrition. Appropriate food formulation could both sustain children’s nutritional needs and favor the acquisition or the proliferation of critical taxa or a community that are present along the digestive tract. But important members of the microbial community that could be favored by the therapeutic food might be partially or completely depleted. In this case, the re-introduction of discrete species or consortia might be needed, in addition to or even in place of food supplements. In the SYNERGIE trial, the probiotic strain *B. infantis* (EVC001) and/or a prebiotic HMO were tested in 2- to 6-month-old Bangladeshi infants suffering from SAM^29^. Daily supplementation of the probiotic or prebiotic increased *B. infantis* abundance in infants with SAM, even if the abundance was 10- to 100-fold lower than in untreated healthy controls. Reduced abundance of Enterobacteriaceae was also observed following supplementation. The probiotic treatment was associated with an increase in weight gain and reduced intestinal inflammation. Preliminary data in gnotobiotic mice suggested that a locally isolated strain (in Bangladesh) *B. infantis Bg_2D9*, could have an even greater fitness impact^29^. Interestingly, infants less than six months old, suffering from SAM, had a higher rate of weight gain when supplemented with the probiotic alone than infants with either the prebiotic or placebo^57^.

Other probiotic strains have also been tested. A meta-analysis of four studies conducted in LMIC concluded that probiotics could improve growth in undernourished children, but studies did not report a lasting effect on the gut microbiome^58^. A study in Uganda followed the evolution of the gut microbiome of infants with SAM from hospital admission to discharge as well as the impact of probiotics during rehabilitation. When admitted to the hospital, children with SAM had a gut microbiome dominated by Proteobacteria, and at discharge, they exhibited increases in Clostridiaceae members. During follow-up, species including *Lactobacillus ruminis*, *Blautia* spp., and *F. prausnitzii* increased, and the microbiome reached similar beta and alpha diversities as healthy children. Interestingly, probiotic supplementation (*L. rhamnosus GG* and *B. animalis* subsp. *lactis BB-12*) reduced the cumulative incidence of diarrhea during the follow-up period compared to children that received a placebo, but only for children in whom the probiotic was detected in the gut^57^. While initial studies are promising, more clinical trials are needed to determine the potential benefits of a range of pre-, pro-, and postbiotics on child nutrition status and health. Of note, in the SHINE trial on 1- to 18-month-old children in rural Zimbabwe, intervention to reduce potential pathogen load, like improvement of water, sanitation, and hygiene (WASH), did not have any effect on the gut microbiome, nor on child growth^39^.

## The microbiome: one of the elements of the vicious circle of undernutrition

Undernutrition seems to have intergenerational origins. Women who were undernourished in childhood have higher odds of delivering a low-birth-weight newborn^59^. Maternal well-being before and during pregnancy can also affect a neonate’s overall development (reviewed in^60^), and some of those effects seem to be mediated by the maternal and child microbiomes^61–63^ (Fig. 1). As an example, in the SHINE study in rural Zimbabwe, gut microbiome composition and metabolic function during pregnancy predicted birth weight and early infant growth (weight-for-age *z*-score) at one month more accurately than gestational age^64^. This intergenerational effect could be mediated by at least three pathways: (1) metabolic transfer during pregnancy, (2) microbiota and metabolite transfer at birth, and (3) then breastfeeding and regular skin contact.

During pregnancy, it is generally believed that the fetal environment (placenta) is sterile^65^. Nevertheless, the maternal gut microbiome secretes a range of metabolites that can enter the maternal circulatory system and are then transported, by way of the placenta, to the fetus^66^. Bacterial metabolites could also impact prenatal development through epigenetic mechanisms^67,68^, for example, by mediating changes in global histone acetylation and methylation, not only in the mother but also in the fetus^69^ (Fig. 1). Only a few studies investigated the impact of undernutrition during pregnancy on infant microbiome and health. In mice, caloric restriction in late pregnancy was associated with a decrease in antimicrobial peptide and protein secretion, suggesting reduced intestinal barrier function and a decreased ability to maintain gut-microbe homeostasis in the mother^70^.

At birth, the neonate is colonized by microbes from its mother via vertical transmission^19^. Accordingly, the transfer of an “altered” microbiome could have consequences on infant microbiome maturation and health. In a study examining how the microbiome affects growth in progeny, mice receiving a microbiome transplant from stunted pups also displayed a stunted phenotype, and this phenotype was transmissible from dams to their offspring. This suggests that a dysbiotic microbiome from an undernourished mother could be transmitted to her infant^47^ (Fig. 1). This seems to also be true for other forms of malnutrition. For example, an obesogenic microbiome transmitted from the mother to the child could have a causal role in inducing postnatal metabolic disorders^71^.

For breastfed infants, the mother milk’s composition, in regards to HMOs and the microbiome, influences infant gut microbiome composition and development. Maternal genetic factors could play a role in milk composition. Carriers of an active fucosyltransferase 2 (FUT2) gene, known as secretors, produce more HMOs, both fucosylated and sialylated^72^. Charbonneau et al.^72^ showed that mothers of stunted infants in Malawi exhibited a significantly lower abundance of HMOs, particularly sialylated HMOs, in breast milk at six months. In the same study, mice and piglets were colonized, by fecal microbiota transplant, with a collection of bacteria from the stool of a child with severe stunting, and then the animals received a Malawian diet with or without supplementation of sialylated bovine milk oligosaccharides (sBMOs) (structurally similar to HMOs). Only mice supplemented with sBMOs exhibited increased weight gain, lean mass, and bone volume. Supplementation solely with inulin (a mixture of fructose polymers similar to fructo-oligosaccharides present in a number of current infant formulas) did not have positive growth effects. In contrast, in a Bangladeshi cohort, a higher relative abundance of sialylated HMOs in mothers’ breast milk was associated with higher odds of SAM^73^. In addition to genetic factors, some data suggest that maternal diet influences human milk composition (HMOs, nutrient profile, and milk microbiome) with a possible impact on child health^74–76^. Together, mounting evidence supports the unique role of HMOs in shaping healthy infant microbiota in early life, and in mediating growth^72,77^.

The microbiome appears as one of the leading factors in the vicious circle of undernutrition, greatly influenced by other genetic, biological, and socio-economic pressures. Thus, the microbiome, among other maternal health factors, may also be considered in more global approaches to break the intergenerational origins of undernutrition. More data from longitudinal, intergenerational studies are still needed.

## Micronutrient deficiencies

Child malnutrition also results from micronutrient deficiencies. Deficiencies in iodine, vitamin A, iron, zinc, calcium, vitamin D, and folate are the most prevalent. Multiple micronutrient powders (MNPs) are designed specifically to address micronutrient deficiencies, including anemia, by improving the quality of children’s diets even when a diverse range of nutritious foods is limited. MNPs are usually introduced as local complementary foods after the age of six months. Interestingly, micronutrients could modulate the gut microbiome or conversely, the gut microbiome could influence dietary micronutrient adequacy.

Vitamin A is critical for the maintenance of intestinal barrier integrity. A study of Bangladeshi neonates who received one vitamin A dose or a placebo within 48 h of birth found that after 6–15 weeks, plasma retinol (vitamin A status) was positively associated with Actinobacteria (the phylum containing *Bifidobacterium*) and *Akkermansia*, but no effect was observed on diversity and abundance of Proteobacteria. An increase of Bifidobacteria after supplementation was significant in boys but not girls^78^.

Iron is required for essential functions and for the generation of effective immune responses^79^. Adequate iron status is therefore a prerequisite for optimal development. A common approach to prevent or treat iron deficiencies is iron fortification and/or supplementation. Data from several models (animal and human) showed that iron supplementation or fortification can affect the gut microbiota^80–84^. Particularly, iron scavenging is key for the survival and growth of bacterial populations. Changes in host iron intake could then modify gut microbiome compositions. Several researchers argue that although iron-containing MNPs are highly effective in reducing iron deficiency anemia, they may increase gastrointestinal morbidity in infants^85^. Indeed, iron can also promote the replication and the virulence of enteric pathogens, such *as Salmonella spp.*, *E. coli* *spp.*, *Shigella* *spp*., and *Campylobacter* *spp*.^80^. Several studies, mostly in Kenya, on six-month-old infants, showed that iron supplementation induced a trend toward increased enteropathogens including *E. coli*, as well as a concurrent decrease in Lactobacillaceae^82–84^. These studies also indicated a significant increase in a biomarker for gut inflammation in the intervention group. It is worth noting that in other Kenyan studies, iron fortification modified the response to broad-spectrum antibiotics and might have reduced their efficacy against potential enteropathogens, particularly pathogenic *E. coli*^86^. Safer formulations might be needed. Iron supplements including zinc^87^, antioxidants such as vitamin E^88^, or prebiotics^89^ could counteract some negative effects of iron supplementation alone. A randomized clinical trial (RCT) on Kenyan children showed that co-provisioning of prebiotic galacto-oligosaccharides (GOS) with MNPs mitigates most of the adverse effects of iron supplementation^89^. GOS targets the *Bifidobacterium* population, supporting the possibility that these bacteria play a central role in the mitigation of the deleterious effects of iron^90^. However, these findings might be context dependent and modulated by other nutritional and or environmental factors. Other RCTs with iron supplementation did not show a negative impact on the gut microbiome^91^. Further understanding of the microbiome has strong potential to help design better and safer interventions against undernutrition and micronutrient deficiencies in at-risk infant populations.

## Conclusions and future perspectives

Undernutrition (with micronutrient deficiencies) is a complex condition driven by several physiological and environmental factors. The microbiome appears to be one of them when its development is impaired. Products or approaches based on microbiome science are promising avenues to improve undernutrition management strategies. But major research questions remain as do challenges related to ethics, sustainability, food safety, and food regulation. New therapeutic foods based on microbiome knowledge could be developed. Results on MDCFs are very encouraging for Bangladeshi children with MAM. It would be important to evaluate if those effects could be generalized to other undernourished child populations around the world or if formulations should be adapted to each sub-population (age, region, urban/rural). More data on how the microbiome is associated with undernutrition in various regions are needed to define a healthy state in each context as exemplified by a study with local probiotic^29^. Biomarkers for the gut microbiome (such as MAZ) could be used for diagnosis but also as indicators or references to evaluate treatment success. Data show that not only gut microbiome markers but the entire digestive tract should be considered, as duodenal and oral microbiomes are uniquely implicated in stunting. In this scenario, less invasive techniques to study the duodenal microbiome are direly needed to ensure the ethical measurement of the small intestine microbiome, to understand its role in undernutrition, and to contribute to the design of new or improved treatments.

Commercially available, ready-to-use MDCFs could be the easiest way for families to administer therapeutic foods to children. Development of such products could be challenging: how should these products be classified and what health claims could be made? The recent evolution of the food regulatory framework might be encouraging. Indeed, international standards for RUTF have been developed at the 45th session of the Codex Alimentarius Commission. RUTFs are now classified as food for special medical purposes. New guidelines might allow future innovations and in particular adoption of local versions of RUTFs by different countries, made affordably, with culturally acceptable ingredients that are locally produced. But it is not clear yet if microbiome-based innovations could be included in this framework. Before they can be used by international agencies, microbiome-supplementing products will need to be evaluated in specific protocols adapted for different populations, taking particular care when examining the infant age class, a class that is directly correlated with gut microbiome maturation. The use of RUTFs and RUSFs for undernutrition management relies on multiple partners’ cooperation (e.g., WHO, United Nations International Children’s Emergency Fund, the World Food Program, Action Contre la Faim, and Doctors Without Borders). Probiotics for undernutrition will face some of the same challenges.

As is currently the case, accessibility will still be a challenge. Some populations will still rely mostly on complementary/family food. In addition, when there is no acute emergency, the use of family food might also be favorable for the development of healthy child-feeding practices, which might not be obtained with ready-to-use formula. In parallel with MDCF developments, more research is strongly needed to evaluate the effect of traditional complementary and family foods on microbiome maturation in various regions and if they can be adapted to better prevent undernutrition in the long term.

Most of the papers discussed here focus on the bacterial players of the microbiota, but children suffering from undernutrition may also carry helminths, protozoa, and diverse fungal populations, which could affect stunting. It was also shown that phage populations are diverse and modulate the gut bacteriome^34^. The contribution of those organisms needs to be better studied as they are thought to be tightly interconnected with bacterial populations and host physiology^92^. It is worth mentioning that other approaches, such as phage therapy, FMTs, and other microbiome-derived products, could be explored in the future. Finally, there is an intergenerational factor in undernutrition that is maintained in part by the transmission of the microbiome to the infant from the mother at birth and through breastfeeding. Maternal health and nutrition, as well as mode of birth and breastfeeding practices, are other elements that need to be considered for holistic and durable prevention of undernutrition.
